# PCR data and comparative performance of *Bacteroidales* microbial source tracking genetic markers

**DOI:** 10.1016/j.dib.2018.04.129

**Published:** 2018-05-05

**Authors:** Pornjira Somnark, Natcha Chyerochana, Akechai Kongprajug, Skorn Mongkolsuk, Kwanrawee Sirikanchana

**Affiliations:** a Applied Biological Sciences, Chulabhorn Graduate Institute, Chulabhorn Royal Academy, Lak Si, Bangkok 10210 Thailand; b Research Laboratory of Biotechnology, Chulabhorn Research Institute, Lak Si, Bangkok 10210 Thailand; c Department of Biotechnology, Faculty of Science, Mahidol University, Bangkok 10400 Thailand; d Center of Excellence on Environmental Health and Toxicology (EHT), Ministry of Education, Bangkok 10210 Thailand

**Keywords:** Endpoint PCR, Fecal pollution, Microbial source tracking, *Bacteroidales*, Sensitivity, Specificity, Water quality

## Abstract

We reported modified endpoint PCR results analyzed by universal and human-, swine-, and cattle-specific *Bacteroidales* gene markers with human sewage and animal fecal samples (i.e., swine, cattle, chicken, goat, sheep, buffalo, and duck) from Tha Chin and Chao Phraya watersheds. Annealing locations of PCR primers were illustrated by maps of 16s rRNA *Bacteroidales* genes. We also summarized previously published work on the performance of the PCR assays. For further discussion of the data presented here, please refer to Somnark et al., Performance evaluation of *Bacteroidales* genetic markers for human and animal microbial source tracking in tropical agricultural watersheds, Environ. Pollut. 236 (2018) 100–110.

**Specifications Table**

**Table:** 

Subject area	*Biology*
More specific subject area	*Applied microbiology*
Type of data	*Tables and figures*
How data were acquired	*PCR instrument (Mastercycler Pro thermocycler, Eppendorf), and literature review*
Data format	*Analyzed*
Experimental factors	*Composite fecal and sewage samples were collected, and DNA extraction was performed*
Experimental features	*PCR primers originally designed as endpoint and quantitative PCR were used in the modified endpoint PCR assays.*
Data source location	*Samples were collected from Tha Chin (Chai Nat, Suphan Buri, Nakhon Pathom, and Samut Sakhon provinces) and Chao Phraya (Phra Nakhon Si Ayutthaya, Pathum Thani, and Bangkok provinces) watersheds, located in the central part of Thailand.*
Data accessibility	*Data are with this article*

**Value of the data**

– PCR results of *Bacteroidales*-modified endpoint PCR markers could be compared with microbial source tracking (MST) studies in other geographic areas for further development of region-specific MST methods.
– *Bacteroidales* PCR primer maps could offer an insight into annealing regions of primers for further design of new primers or evaluating currently available primers with their performance.
– A summary of PCR assays that are originally designed and adopted to other regions could serve as a database for comparing the MST method performance in different geographical areas.

## 1 Data

**Fig. 1:**
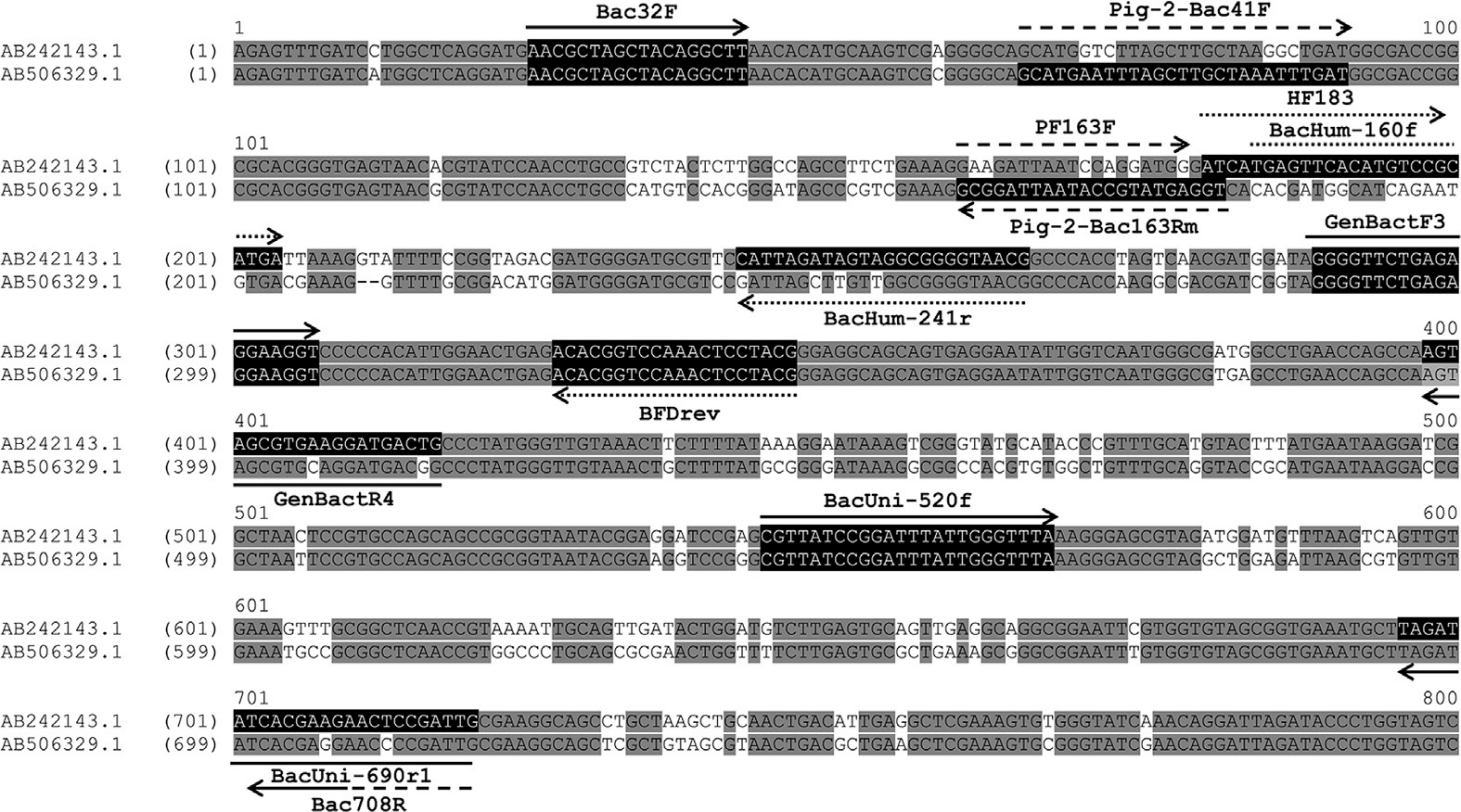
Primer map targeting the 16 S rRNA gene of human- and swine-associated *Bacteroidales*. All primers were BLASTed against the NCBI database. The representative sequences from human feces (Accession no. AB242143.1 [24]) and swine feces (AB506329.1 [25]) were selected to align with specific primers. Human-specific, swine-specific and universal *Bacteroidales* primers are indicated in dotted, dashed and solid arrows, respectively.

**Fig. 2:**
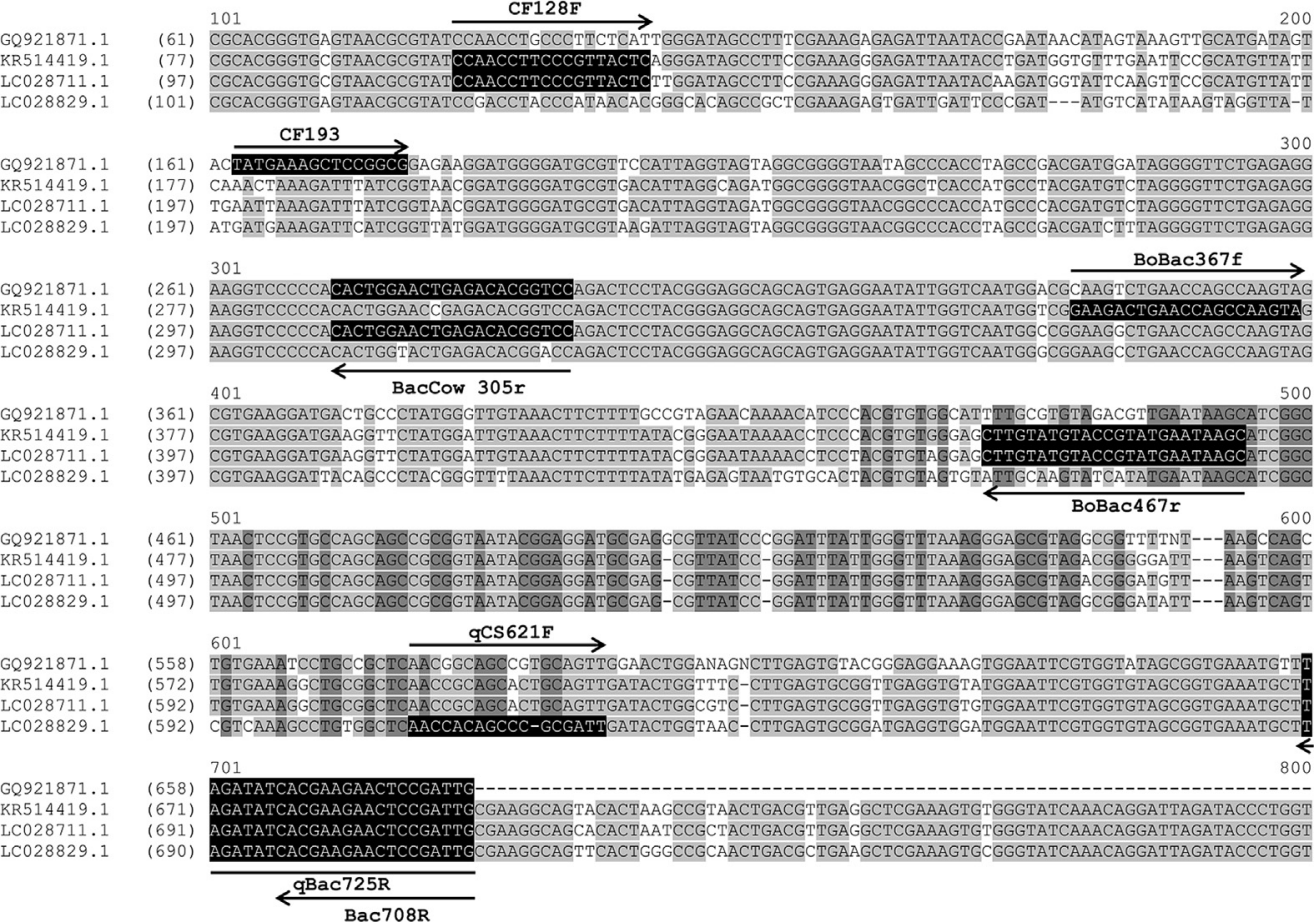
Primer map targeting the 16 S rRNA gene of cattle-associated *Bacteroidales*. All primers were BLASTed against the NCBI database. The representative sequences (Accession nos. GQ921871.1 [26], KR514419.1, LC028711.1, and LC028829.1) were selected to align with specific primers.

**Fig. 3:**
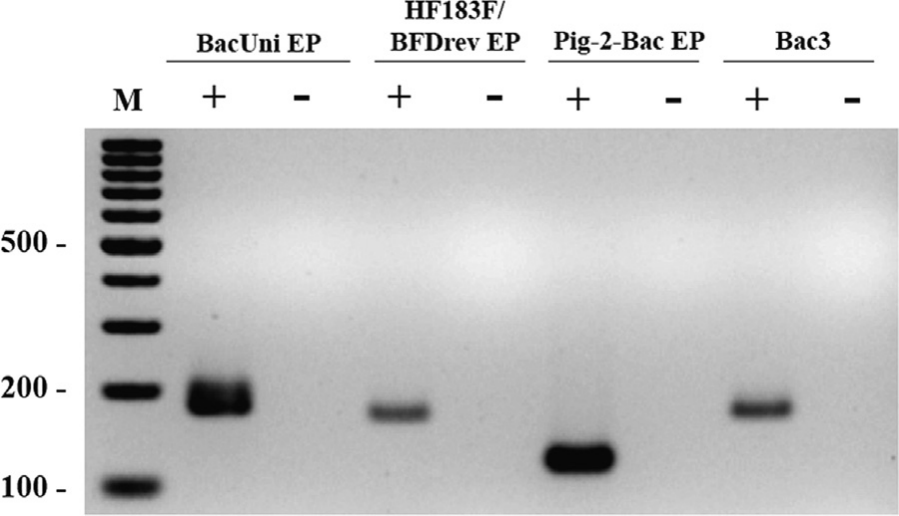
PCR results showing amplification products for universal and human-, swine-, and cattle-specific *Bacteroidales* markers (see [2] for related information).

We performed endpoint PCR assays modified from published methods originally in PCR and qPCR platforms. PCR results of ten good-performing modified endpoint PCR assays against human sewage and animal fecal samples from Tha Chin and Chao Phraya watersheds are shown (Table 1). There were six modified endpoint PCR assays that demonstrated potentially low sensitivity or specificity during the process of testing against a limited number of samples and therefore were not further tested with total samples (Table 2). We also compiled sensitivity and specificity data of previously published *Bacteroidales* genetic markers from both studies that originally designed the assays and studies that adopted the designed assays to be used in another geographic location (Table 3). To provide further insight into PCR performance, we mapped PCR primers to 16 s rRNA gene of human-, swine-, and cattle-associated *Bacteroidales* (Fig. 1, Fig. 2). Amplified PCR products with universal and human-, swine-, and cattle-specific *Bacteroidales* PCR assays were presented (Fig. 3).

**Table 1:** Positive PCR results of modified endpoint PCR markers showing good performance with samples from Tha Chin and Chao Phraya watersheds.

Host	Assay name	Tha Chin watershed								Chao Phraya watershed					
		Human (19)	Swine (20)	Cattle (20)	Chicken (19)	Goat (7)	Sheep (5)	Buffalo (5)	Duck (5)	Human (9)	Swine (8)	Cattle (5)	Chicken (2)	Goat (3)	Buffalo (1)
Universal	BacUni EP	19	20	20	20	7	5	5	5	9	8	5	1	3	1
	GenBac3 EP	19	20	20	20	7	5	5	5	9	8	5	2	3	1
	Bac32F/Bac708R	15	20	20	20	7	5	5	5	9	8	5	2	3	1
															
Human	BacHum EP	18	17	1	9	5	2	1	2	9	7	0	0	2	0
	HF183F/BFDrev EP	16	4	1	9	2	1	0	2	9	0	0	0	0	0
	Modified HF183F/Bac708R	18	4	4	12	1	1	0	2	9	5	0	0	1	0
Swine	Pig-2-Bac EP	0	20	0	2	0	0	0	0	0	8	0	0	0	0
															
Cattle	Bac2	0	0	14	0	0	0	0	0	0	0	5	0	0	0
	Bac3	0	0	17	0	0	0	0	0	0	0	5	0	0	0
	Cow-Bac2 EP	0	1	18	18	7	5	5	4	0	0	5	1	3	1

**Table 2:** Positive PCR results of modified endpoint PCR markers showing relatively poor performance with limited numbers of samples from the Tha Chin watershed.

Fecal origin	Assay name	No. of positive samples/no. of samples tested^a^								Sensitivity	Specificity	Accuracy
		Human	Swine	Cattle	Chicken	Goat	Sheep	Buffalo	Duck			
Swine	PF163F/Bac708R	0/0	20/20	6/20	3/19	0/7	1/5	4/5	0/5	1.00	0.77	0.83
												
Cattle	CowM2 EP	0/0	3/3	6/7	1/1	2/2	0/0	0/0	0/0	0.86	0.00	0.47
	BacCow EP	0/0	3/3	7/7	1/1	2/2	0/0	0/0	0/0	1.00	0.00	0.54
	CF193F/Bac708R	0/0	0/3	0/7	0/1	0/2	0/0	0/0	0/0	0.00	1.00	0.46
												
	CF128F/Bac708R	0/0	3/3	7/7	1/1	2/2	0/0	0/0	0/0	1.00	0.00	0.54
											
	BoBac EP	0/0	18/19	20/20	0/0	0/0	0/0	0/0	0/0	1.00	0.05	0.54

a Limited number of animal samples tested for certain assays due to potentially low sensitivity or specificity.

**Table 3:** Sensitivity and specificity of *Bacteroidales* markers in studied in which the assays were originally designed and adopted to other geographic regions.

**Host source**	**Assay name**	**Platform**	**Geographical region**	**Original/ Adopted**	**Sensitivity (n)**^a^	**Specificity (n)**	**Non-target hosts**	**Reference**
Universal	BacUni	qPCR	California, USA	Original	1.00 (n=73)	NA^b^	Human^c^, cow, horse, dog, cat, seagull, WWTP^d^ (primary influent)	[1]
		PCR	Tha Chin watershed, Thailand	Adopted	1.00 (n=100, composite)	NA	Swine, cattle, chicken, goat, sheep, buffalo, duck, sewage^e^	[2]
		PCR	Chao Phraya watershed, Thailand	Adopted	0.96 (n=28, composite)	NA	Swine, cattle, chicken, goat, buffalo, sewage	[2]
								
Universal	GenBac3	qPCR	Louisiana, Michigan, Mississippi, USA	Original	NA	NA	Surface water sample	[3]
						
		PCR	Tha Chin watershed, Thailand	Adopted	1.00 (n=100, composite)	NA	Swine, cattle, chicken, goat, sheep, buffalo, duck, sewage	[2]
			Chao Phraya watershed, Thailand	Adopted	1.00 (n=28, composite)	NA	Swine, cattle, chicken, goat, buffalo, sewage	[2]
Universal	Bac32F/Bac708R	PCR	Oregon, USA	Original	1.00 (n=30)	NA	Human, cow	[4]
		PCR	Southeast Queensland, Australia	Adopted	1.00 (n=186)	NA	Cattle, pig, sheep, goat, horse, chicken, dog, duck, pelican, kangaroo, WWTP	[5] (one base pair mismatch for Bac32F primer)
		PCR	Wisconsin, USA	Adopted	1.00 (n=89)	NA	Cow, WWTP	[6]
		PCR	Missouri, USA	Adopted	0.89 (n=286)	NA	Human, sewage, dog, beef cattle, dairy cattle, chicken, turkey, horse, swine, goose	[7]
		PCR	Britanny and Normandy, France	Adopted	0.96 (n=136)	NA	Pig, cow, sheep, chicken, wild bird	[8]
		PCR	Saskatchewan, Canada	Adopted	1.00 (n=273)	NA	Human, WWTP, cow, pig, chicken, goose, moose, deer, caribou, bison, goat	[9]
		PCR	Illinois, Nebraska, Ohio, Texas, Delaware, and West Virginia, USA	Adopted	0.78 (n=222)	NA	Cattle, human, chicken, raccoon, horse, pig, pig manure pit, pig waste lagoon	[10]
		PCR	Puerto Rico, USA	Adopted	0.89 (n=356)	NA	Cow, goat, horse, swine, monkey, fish, pigeon, chicken, guinea fowl, duck, turkey, swan, WWTP	[11]
								
		PCR	Tha Chin watershed, Thailand	Adopted	0.96 (n=100, composite)	NA	Swine, cattle, chicken, goat, sheep, buffalo, duck, sewage	[2]
					
			Chao Phraya watershed, Thailand	Adopted	1.00 (n=28, composite)	NA	Swine, cattle, chicken, goat, buffalo, sewage	[2]
								
Human	BacHum	qPCR	California, USA	Original	0.67 (n=18);	0.98 (n=41)	Cow, horse, dog, cat, seagull	[1]
					1.00 (n=14, sewage)			
		PCR	Southeast Queensland, Australia	Adopted	1.00 (n=50, WWTP)	0.96 (n=136)	Cattle, pig, sheep, goat, horse, chicken, dog, duck, pelican, kangaroo	[5]
								
		PCR	Tha Chin watershed, Thailand	Adopted	0.95 (n=19, sewage)	0.54 (n=81, composite)	Swine, cattle, chicken, goat, sheep, buffalo, duck	[2]
					
			Chao Phraya watershed, Thailand	Adopted	1.00 (n=9, sewage)	0.53 (n=19, composite)	Swine, cattle, chicken, goat, buffalo	[2]
Human	HF183/BFDrev	qPCR	Michigan, Minnesota, Colorado, South Dakota, Wyoming, Hawaii, Virginia, Ohio, Florida, North Carolina, and New York, USA	Original	1.00 (n=14, WWTP)	0.60 (n=5, composite)	Cow, pig, chicken, dog, cat	[12]
								
		PCR	Tha Chin watershed, Thailand	Adopted	0.84 (n=19, sewage)	0.77 (n=81, composite)	Swine, cattle, chicken, goat, sheep, buffalo, duck	[2]
					
			Chao Phraya watershed, Thailand	Adopted	1.00 (n=9, sewage)	1.00 (n=19, composite)	Swine, cattle, chicken, goat, buffalo	[2]
Human	HF183/Bac708R	PCR	Oregon, USA	original	0.85 (n=13); 1.00 (n=3, WWTP)	1.00 (n=46)	Cow, deer, elk, cat, dog, duck, pig, gull, goat, llama, sheep	[13]
		PCR	Southeast Queensland, Australia	Adopted	1.00 (n=52, WWTP)	1.00 (n=155)	Duck, kangaroos, cattle, horse, dog, chicken, pig, pelican, goat, deer, wild birds, sheep	[14]
		PCR	Spain	Adopted	0.50 (n=40, WWTP)	0.71 (n=73)	Poultry, pig, cow	[15]
		PCR	Southeast Queensland, Australia	Adopted	1.00 (n=59, WWTP); 0.80 (n=20)	0.95 (n=214)	Bird, camel, cattle, chicken, dog, duck, horse, kangaroo, pig, possom	[16]
		PCR	Britanny and Normandy, France	Adopted	0.98 (n=44)	0.99 (n=86)	Pig, cow, sheep, chicken, wild bird	[8]
		PCR	Puerto Rico, USA	Adopted	0.75 (n=16, sewage WWTP)	1.00 (n=340)	Cow, goat, horse, swine, monkey, fish, pigeon, chicken, guinea fowl, duck, turkey, swan	[11]
		PCR	Wisconsin, USA	Adopted	1.00 (n=14, WWTP)	1.00 (n=75)	Cow	[6]
								
		PCR	Saskatchewan, Canada	Adopted	1.00 (n=8, WWTP);	1.00 (n=211)	Cow, pig, chicken, goose, moose, deer, caribou, bison, goat	[9]
					0.94 (n=54)			
								
		PCR	Tha Chin watershed, Thailand	Adopted	0.95 (n=19, sewage)	0.70 (n=81, composite)	Swine, cattle, chicken, goat, sheep, buffalo, duck	[2]
					
			Chao Phraya watershed, Thailand	Adopted	1.00 (n=9, sewage)	0.68 (n=19, composite)	Swine, cattle, chicken, goat, buffalo	[2]
Swine	PF163F/Bac708R	PCR	Cincinnati, Ohio	Original	1.00 (n=19)	NA	NA	[17]
		PCR	Saskatchewan, Canada	Adopted	1.00 (n=50)	1.00 (n=223)	Human, WWTP, cow, chicken, goose, moose, deer, caribou, bison, goat	[9]
		PCR	Illinois, Nebraska, Ohio, Texas, Delaware, and West Virginia, USA	Adopted	0.87 (n=97); 1.00 (n=6, slurry)	0.77 (n=119)	Cattle, cattle lagoon, human, chicken, raccoon, horse	[10]
		PCR	Puerto Rico, USA	Adopted	1.00 (n=30)	0.75 (n=261)	Cow, goat, horse, monkey, fish, pigeon, chicken, guinea fowl, duck, turkey, swan, WWTP	[11]
		PCR	Britanny and Normandy, France	Adopted	1.00 (n=25)	0.98 (n=105)	Human, cow, sheep, chicken, wild bird	[8]
		PCR	Tha Chin watershed, Thailand	Adopted	1.00 (n=20, composite)	0.77 (n=61, composite)	Cattle, chicken, goat, sheep, buffalo, duck	[2]
								
Swine	Pig-2-Bac	qPCR	Brittany, France	Original	1.00 (n=25);	1.00 (n=54)	Human, bovine, horse, sheep	[18]
					1.00 (n=23, slurry)			
								
		PCR	Tha Chin watershed, Thailand	Adopted	1.00 (n=20, composite)	0.98 (n=80, composite)	Cattle, chicken, goat, sheep, buffalo, duck, sewage	[2]
					
			Chao Phraya watershed, Thailand	Adopted	1.00 (n=8, composite)	1.00 (n=20, composite)	Cattle, chicken, goat, buffalo, sewage	[2]
Cattle	CowM2	qPCR	West Virginia, Georgia, Wyoming, Delaware, Florida, and Ohio, USA	Original	1.00 (n=60)	1.00 (n=139); 1.00 (n=5, WWTP (primary effluent)	Alpaca, goat, mule deer, sheep, Canadian goose, cat, chicken, dog, duck, horse, human, pelican, pig, sea gull, turkey	[19]
		PCR	Tha Chin watershed, Thailand	Adopted	0.86 (n=7, composite)	0.00 (n=6, composite)	Swine, chicken, goat	[2]
								
Cattle	BacCow	qPCR	California, USA	Original	1.00 (n=8)	0.95 (n=65)	Human, horse, dog, cat, seagull, WWTP (primary effluent)	[1]
		PCR	Tha Chin watershed, Thailand	Adopted	1.00 (n=7, composite)	0.00 (n=6, composite)	Swine, chicken, goat	[2]
								
Cattle	CF193/Bac708R	PCR	Oregon, USA	Original	1.00 (n=19)	0.72 (n=43)	Human, WWTP, deer, elk, cat, dog, duck, pig, gull, goat, llama, sheep	[13]
		PCR	Wisconsin, USA	Adopted	0.85 (n=75)	NA	NA	[6]
		PCR	Saskatchewan, Canada	Adopted	0.16 (n=32)	NA	NA	[9]
		PCR	Spain, UK, Cyprus, France, and Sweden	Adopted	0.00 (n=19, ruminant)	0.99 (n=94)	WWTP, poultry, pig	[15]
		PCR	USA	Adopted	0.68 (n=247)	1.00 (n=175)	Alpaca, pronghorn, elk, gazelle, giraffe, goat, mule deer, okapi, sheep, takin, tufted deer, moose, white-tailed deer, Canadian goose, cat, chicken, dog, duck, horse, human, pelican, pig, raccoons, sea gull, turkey	[20]
		PCR	Tha Chin watershed, Thailand	Adopted	0.00 (n=7, composite)	1.00 (n=6, composite)	Swine, chicken, goat	[2]
								
Cattle	CF128F/Bac708R	PCR	Oregon, USA	Original	1.00 (n=19)	0.77 (n=43)	Human, WWTP, deer, elk, cat, dog, duck, pig, gull, goat, llama, sheep	[13]
		PCR	Wisconsin, USA	Adopted	1.00 (n=75)	0.93 (n=14)	WWTP	[6]
		PCR	Britanny and Normandy, France	Adopted	1.00 (n=32)	0.60 (n=98)	Human, pig, chicken, sheep, wild bird	[8]
								
		PCR	Saskatchewan, Canada	Adopted	0.96 (n=51, cow);	0.62 (n=222, cow);	Human, WWTP, pig, chicken, goose	[9]
	
					0.98 (n=121, ruminant=cow, deer, caribou, bison, moose, goat)	0.93 (n=152, ruminant=cow, deer, caribou, bison, moose, goat)		
		PCR	Spain	Adopted	0.26 (n=19, ruminant)	1.00 (n=95)	WWTP, poultry, pig	[15]
		PCR	USA	Adopted	0.85 (n=247)	0.76 (n=175)	Alpaca, pronghorn, elk, gazelle, giraffe, goat, mule deer, okapi, sheep, takin, tufted deer, moose, white-tailed deer, Canadian goose, cat, chicken, dog, duck, horse, human, pelican, pig, raccoons, sea gull, turkey	[20]
		PCR	Puerto Rico, USA	Adopted	0.64 (n=66)	0.90 (n=290)	Goat, horse, swine, monkey, fish, pigeon, chicken, guinea fowl, duck, turkey, swan, WWTP	[11]
		PCR	Tha Chin watershed, Thailand	Adopted	1.00 (n=7, composite)	0.00 (n=6, composite)	Swine, chicken, goat	[2]
								
Cattle	Bac2	PCR	USA	Adopted	0.54 (n=148)	1.00 (n=279)	Bird, human, domestic, wildlife, pets, water by cattle	[21]
		PCR	USA	Adopted	0.54 (n=247)	1.00 (n=175)	Alpaca, pronghorn, elk, gazelle, giraffe, goat, mule deer, okapi, sheep, takin, tufted deer, moose, white-tailed deer, Canadian goose, cat, chicken, dog, duck, horse, human, pelican, pig, raccoons, sea gull, turkey	[20]
								
		PCR	Tha Chin watershed, Thailand	Adopted	0.70 (n=20, composite)	1.00 (n=80, composite)	Swine chicken, goat, sheep, buffalo, duck, sewage	[2]
					
			Chao Phraya watershed, Thailand	Adopted	1.00 (n=5, composite)	1.00 (n=23, composite)	Swine chicken, goat, buffalo, sewage	[2]
Cattle	Bac3	PCR	USA	Original	0.91 (n=148)	0.99 (n=245)	Human, sewage, bovine, chicken, black vulture, Canadian goose, peacock, pigeon, dog, cat, guinea pig, domestic goat, pig, sheep, horse, alpaca, llama, armadillo, bobcat, coyote, gray squirrel, rabbit, opossum, raccoon, whitetail deer, wild turkey, hedgehog, prairie dog	[21]
		PCR	USA	Adopted	0.69 (n=247, ind)	0.99 (n=175, ind)	Alpaca, pronghorn, elk, gazelle, giraffe, goat, mule deer, okapi, sheep, takin, tufted deer, moose, white-tailed deer, canadian goose, cat, chicken, dog, duck, horse, human, pelican, pig, raccoons, sea gull, turkey	[20]
								
		PCR	Tha Chin watershed, Thailand	Adopted	0.85 (n=20, composite)	1.00 (n=80, composite)	Swine chicken, goat, sheep, buffalo, duck, sewage	[2]
					
			Chao Phraya watershed, Thailand	Adopted	1.00 (n=5, composite)	1.00 (n=23, composite)	Swine chicken, goat, buffalo, sewage	[2]
Cattle	Cow-Bac2	qPCR	Sapporo and Ebetsu Cities, Japan	Original	1.00 (n=7)	1.00 (n=9)	Human, pig	[22]
								
		PCR	Tha Chin watershed, Thailand	Adopted	0.90 (n=20, composite)	0.50 (n=80, composite)	Swine chicken, goat, sheep, buffalo, duck, sewage	[2]
					
			Chao Phraya watershed, Thailand	Adopted	1.00 (n=5, composite)	0.78 (n=23, composite)	Swine chicken, goat, buffalo, sewage	[2]
Cattle	BoBac	qPCR	Tennessee, Pennsylvania, and Texas, USA	Adopted	1.00 (n=11)	0.87 (n=15)	Human, swine, canine, equine	[23]
		PCR	Tha Chin watershed, Thailand	Adopted	1.00 (n=20, composite)	0.05 (n=19, composite)	Swine	[2]

a Total number of samples being tested.

b Not applicable.

c Human individual fecal sample.

d Influent of municipal wastewater treatment plant, unless stated otherwise.

e Influent of wastewater treatment system in buildings or septic tanks.

## 2 Experimental design, materials and methods

### 2.1 Sample collection and DNA extraction

Raw human sewage and non-human fecal samples were collected from Tha Chin and Chao Phraya watersheds. One composite fecal sample was prepared by mixing fresh feces of at least 20 individuals. Samples were transported on ice to the laboratory. DNA extraction of composite fecal samples and 0.22-µm-pore-size mixed cellulose ester membrane (Merck Millipore, Billerica, MA, USA) after 50–100 mL human sewage filtration was performed with a ZR Fecal DNA MiniPrep kit (Zymo Research, Irvine, CA, USA). DNA concentrations were measured using a NanoDrop spectrophotometer (Thermo Scientific, Wilmington, DE, USA).

### 2.2 PCR method modification and performance criteria

PCR primers targeting universal and human-, swine-, and cattle-specific fecal markers were selected from both endpoint and quantitative PCR platforms (Table 4). A 10-μL PCR is composed of 0.5 μL each of 10 μM forward primers and 10 μM reverse primers, 1-μL of DNA template (corresponding to 0.2, 2.0 or 20 ng total DNA), 5 μL of DreamTaq PCR Master Mix (2×; Thermo Fisher Scientific, Waltham, MA, USA), and sterile water. The reaction was processed in a Mastercycler Pro thermocycler (Eppendorf, Hamburg, Germany). PCR cycling conditions were modified as follows: initial denaturation at 95 °C for 3 min; 30 cycles of a denaturation step at 95 °C for 30 s, an annealing step at varying temperature and time (Table 4), and an elongation step at 72 °C for 30 s; and a final extension at 72 °C for 10 min. PCR products were visualized with a Gel Doc XR system (BIO-RAD, Hercules, CA, USA). PCRs were run in duplicate. No-template controls and extraction blanks were included for quality control. Performance criteria including sensitivity, specificity, and accuracy were calculated as TP/(TP+FN), TN/(TN+FP), and (TP+TN)/(TP+FP+TN+FN), respectively, where TP, FN, TN, and FP, are true positive, false negative, true negative, and false positive, respectively.

**Table 4:** Primer sequences and PCR cycling conditions.

Host	Assay name	Primer name	Primer sequence (5′ - 3′)	Annealing temperature (°C)	Annealing time (s)	Original platform	Reference
Universal	BacUni EP	BacUni-520f	CGT-TAT-CCG-GAT-TTA-TTG-GGT-TTA	60.0	30	qPCR	[1]
		BacUni-690r1	CAA-TCG-GAG-TTC-TTC-GTG-ATA-TCT-A				
	GenBac3 EP	GenBac3F	GGG-GTT-CTG-AGA-GGA-AGG-T	60.0	30	qPCR	[3]
		GenBac3R	CCG-TCA-TCC-TTC-ACG-CTA-CT				
	Bac32F/Bac708R	Bac32F	AAC-GCT-AGC-TAC-AGG-CTT	53.7	60	PCR	[4], [27]
		Bac708R	CAA-TCG-GAG-TTC-TTC-GTG				
Human sewage	BacHum EP	BacHum-160f	TGA-GTT-CAC-ATG-TCC-GCA-TGA	60.0	30	qPCR	[1]
		BacHum-241r	CGT-TAC-CCC-GCC-TAC-TAT-CTA-ATG				
	HF183/BFDrev EP	HF183	ATC-ATG-AGT-TCA-CAT-GTC-CG	60.0	30	qPCR	[12]
		BFDrev	CGT-AGG-AGT-TTG-GAC-CGT-GT				
	Modified HF183F/Bac708R	HF183F	ATC-ATG-AGT-TCA-CAT-GTC-CG	55.3	60	PCR	[13], [27]
		Bac708R	CAA-TCG-GAG-TTC-TTC-GTG				
Swine	PF163F/Bac708R	PF163F	GCG-GAT-TAA-TAC-CGT-ATG-A	52.4	60	PCR	[17], [27]
		Bac708R	CAA-TCG-GAG-TTC-TTC-GTG				
	Pig-2-Bac EP	Pig-2-Bac41F	GCA-TGA-ATT-TAG-CTT-GCT-AAA-TTT-GAT	60.0	30	qPCR	[18]
		Pig-2-Bac163Rm	ACC-TCA-TAC-GGT-ATT-AAT-CCG-C				
Cattle	CowM2 EP	CowM2F	CGG-CCA-AAT-ACT-CCT-GAT-CGT	60.0	30	qPCR	[19]
		CowM2R	GCT-TGT-TGC-GTT-CCT-TGA-GAT-AAT				
	BacCow EP	CF128F	CCA-ACY-TTC-CCG-WTA-CTC	60.0	30	qPCR	[1]
		BacCow 305r	GGA-CCG-TGT-CTC-AGT-TCC-AGT-G				
	CF193F/Bac708R	CF193	TAT-GAA-AGC-TCC-GGC-C	55.0	30	PCR	[13]
		Bac708R	CAA-TCG-GAG-TTC-TTC-GTG				
	Modified CF128F/Bac708R	CF128F	CCA-ACY-TTC-CCG-WTA-CTC	62.0	60	PCR	[13], [28]
		Bac708R	CAA-TCG-GAG-TTC-TTC-GTG				
	Bac2	Bac2F	GCT-TGT-TGC-GTT-CCT-TGAGAT-AAT	62.0	30	PCR	[21]
		Bac2R	ACA-AGC-CAG-GTG-ATA-CAG-AAA-G				
	Bac3	Bac3F	CTA-ATG-GAA-AAT-GGA-TGG-TAT-CT	60.0	30	PCR	[21]
		Bac3R	GCC-GCC-CAG-CTC-AAA-TAG				
	Cow-Bac2 EP	qCS621F	AAC-CAC-AGC-CCG-CGA-TT	62.0	30	SYBR qPCR	[22]
		qBac725R	CAA-TCG-GAG-TTC-TTC-GTG-ATA-TCT-A				
	BoBac EP	BoBac367f	GAA-GAC-TGA-ACC-AGC-CAA-GTA	57.0	30	qPCR	[23]
		BoBac467r	GCT-TAT-TCA-TAC-GGT-ACA-TAC-AAG				

## References

[1] Kildare B.J., Leutenegger C.M., McSwain B.S., Bambic D.G., Rajal V.B., Wuertz S. (2007). 16S rRNA-based assays for quantitative detection of universal, human-, cow-, and dog-specific fecal *Bacteroidales:* a Bayesian approach. Water Res..

[2] Somnark P., Chyerochana N., Mongkolsuk S., Sirikanchana K. (2018). Performance evaluation of *Bacteroidales* genetic markers for human and animal microbial source tracking in tropical agricultural watersheds. Environ. Pollut..

[3] Siefring S., Varma M., Atikovic E., Wymer L., Haugland R.A. (2008). Improved real-time PCR assays for the detection of fecal indicator bacteria in surface waters with different instrument and reagent systems. J. Water Health..

[4] Bernhard A.E., Field K.G. (2000). Identification of nonpoint sources of fecal pollution in coastal waters by using host-specific 16S ribosomal DNA genetic markers from fecal anaerobes. Appl. Environ. Microbiol..

[5] Ahmed W., Goonetilleke A., Powell D., Gardner T. (2009). Evaluation of multiple sewage-associated *Bacteroides* PCR markers for sewage pollution tracking. Water Res..

[6] Bower P., Scopel C.O., Jensen E.T., Depas M.M., Mclellan S.L. (2005). Detection of genetic markers of fecal indicator bacteria in Lake Michigan and determination of their relationship to *Escherichia coli* densities using standard microbiological methods. Appl. Environ. Microbiol..

[7] Carson C.A., Christiansen J.M., Benson V.W., Baffaut C., Jerri V., Broz R.R., Kurtz W.B., Rogers W.M., Fales W.H., Yampara-iquise H., Davis J.V. (2005). Specificity of a *Bacteroides the taiotaomicron* marker for human feces. Appl. Environ. Microbiol..

[8] Gourmelon M., Caprais M.P., Segura R., Le Mennec C., Lozach S., Piriou J.Y., Rince A. (2007). Evaluation of two library-independent microbial source tracking methods to identify sources of fecal contamination in French estuaries. Appl. Environ. Microbiol..

[9] Fremaux B., Gritzfeld J., Boa T., Yost C.K. (2009). Evaluation of host-specific *Bacteroidales* 16S rRNA gene markers as a complementary tool for detecting fecal pollution in a prairie watershed. Water Res..

[10] Lamendella R., Santo Domingo J.W., Yannarell A.C., Ghosh S., Di Giovanni G., Mackie R.I., Oerther D.B. (2009). Evaluation of swine-specific PCR assays used for fecal source tracking and analysis of molecular diversity of swine-specific “*Bacteroidales*” populations. Appl. Environ. Microbiol..

[11] Toledo-Hernandez C., Ryu H., Gonzalez-Nieves J., Huertas E., Toranzos G., Domingo J.W. Santo (2013). Tracking the primary sources of fecal pollution in a tropical watershed in a one-year study. Appl. Environ. Microbiol..

[12] Haugland R.A., Varma M., Sivaganesan M., Kelty C., Peed L., Shanks O.C. (2010). Evaluation of genetic markers from the 16S rRNA gene V2 region for use in quantitative detection of selected *Bacteroidales* species and human fecal waste by qPCR. Syst. Appl. Microbiol..

[13] Bernhard A.E., Field K.G., PCR A. (2000). assay to discriminate human and ruminant feces on the basis of host differences in *Bacteroides-Prevotella* genes encoding 16S rRNA. Appl. Environ. Microbiol..

[14] Ahmed W., Stewart J., Powell D., Gardner T. (2008). Evaluation of *Bacteroides* markers for the detection of human faecal pollution. Lett. Appl. Microbiol.

[15] Ballesté E., Bonjoch X., Belanche L.A., Blanch A.R. (2010). Molecular indicators used in the development of predictive models for microbial source tracking. Appl. Environ. Microbiol..

[16] Ahmed W., Masters N., Toze S. (2012). Consistency in the host specificity and host sensitivity of the *Bacteroides* HF183 marker for sewage pollution tracking. Lett. Appl. Microbiol.

[17] Dick L.K., Bernhard A.E., Brodeur T.J., Santo Domingo J.W., Simpson J.M., Walters S.P., Field K.G. (2005). Host distributions of uncultivated fecal *Bacteroidales* bacteria reveal genetic markers for fecal source identification. Appl. Environ. Microbiol..

[18] Mieszkin S., Furet J.P., Corthier G., Gourmelon M. (2009). Estimation of pig fecal contamination in a river catchment by real-time PCR using two pig-specific *Bacteroidales* 16S rRNA genetic markers. Appl. Environ. Microbiol..

[19] Shanks O.C., Atikovic E., Blackwood A.D., Lu J., Noble R.T., Domingo J.S., Seifring S., Sivaganesan M., Haugland R.A. (2008). Quantitative PCR for detection and enumeration of genetic markers of bovine fecal pollution. Appl. Environ. Microbiol..

[20] Shanks O.C., White K., Kelty C.A., Hayes S., Sivaganesan M., Jenkins M., Varma M., Haugland R.A. (2010). Performance assessment PCR-based assays targeting *Bacteroidales* genetic markers of bovine fecal pollution. Appl. Environ. Microbiol..

[21] Shanks O.C., Santo Domingo J.W., Lamendella R., Kelty C.A., Graham J.E. (2006). Competitive metagenomic DNA hybridization identifies host-specific microbial genetic markers in cow fecal samples. Appl. Environ. Microbiol..

[22] Okabe S., Okayama N., Savichtcheva O., Ito T. (2007). Quantification of host-specific *Bacteroides-Prevotella* 16S rRNA genetic markers for assessment of fecal pollution in freshwater. Appl. Microbiol. Biotechnol..

[23] Layton A., McKay L., Williams D., Garrett V., Gentry R., Sayler G. (2006). Development of *Bacteroides* 16S rRNA gene taqman-based real-time PCR assays for estimation of total, human, and bovine fecal pollution in water. Appl. Environ. Microbiol..

[24] Bakir M.A., Sakamoto M., Kitahara M., Matsumoto M., Benno Y. (2006). *Bacteroides dorei* sp. nov., isolated from human faeces. Int. J. Syst. Evol. Microbiol..

[25] Kobayashi Y., Itoh A., Miyawaki K., Koike S., Iwabuchi O., Iimura Y., Kobashi Y., Kawashima T., Wakamatsu J., Hattori A., Murakami H., Morimatsu F., Nakaebisu T., Hishinuma T. (2011). Effect of liquid whey feeding on fecal microbiota of mature and growing pigs. Anim. Sci. J.

[26] Jeong J.Y., Park H.D., Lee K.H., Hwang J.H., Ka J.O. (2010). Quantitative analysis of human- and cow-specific 16S rRNA gene markers for assessment of fecal pollution in river waters by real-time PCR. J. Microbiol. Biotechnol..

[27] Hussein K.R., Waines P.L., Nisr R.B., Glegg G., Bradley G. (2014). Development and use of *Bacteroides* 16S rRNA polymerase chain reaction assay for source tracking dog faecal pollution in bathing waters. Hydrol.: Curr. Res..

[28] Lamendella R., Domingo J.W.S., Oerther D.B., Vogel J.R., Stoeckel D.M. (2007). Assessment of fecal pollution sources in a small northern-plains watershed using PCR and phylogenetic analyses of *Bacteroidetes* 16S rRNA gene. FEMS Microbiol. Ecol..

