# Supplementary material for: PCR data and comparative performance of Bacteroidales microbial source tracking genetic markers
Source: Data Brief. 2018 May 5;19:156–69. doi: 10.1016/j.dib.2018.04.129 (PMC5992956; doi:10.1016/j.dib.2018.04.129)
Supplement: Supplementary file 1 — Supplementary material [file mmc1.pdf]

Conflicts of Interest Statement

Manuscript title: PCR data and comparative performance of Bacteroidales microbial source tracking genetic markers

The authors whose names are listed immediately below certify that they have NO affiliations with or involvement in any organization or entity with any financial interest (such as honoraria; educational grants; participation in speakers' bureaus; membership, employment, consultancies, stock ownership, or other equity interest; and expert testimony or patent-licensing arrangements), or non-financial interest (such as personal or professional relationships, affiliations, knowledge or beliefs) in the subject matter or materials discussed in this manuscript.

Author names:

Pornjira Somnark

Natcha Chyerochana

Akechai Kongprajug

Skorn Mongkolsuk

Kwanrawee Sirikanchana

The authors whose names are listed immediately below report the following details of affiliation or involvement in an organization or entity with a financial or non-financial interest in the subject matter or materials discussed in this manuscript. Please specify the nature of the conflict on a separate sheet of paper if the space below is inadequate.

Author names:

Scanned by CamScanner

This statement is signed by all the authors to indicate agreement that the above information is true and correct (*a photocopy of this form may be used if there are more than 10 authors*):

Author's name (typed)	Author's signature	Date
Pornjira Somnark	พรจิรา สมนาค .	Feb 8, 2018
Natcha Chyerochana	[illegible]	Feb 8, 2018
Akechai Kongprajug	Kongprajug A.	Feb 8, 2018
Skorn Mongkolsuk	[illegible]	Feb 8, 2018
Kwanrawee Sirikanchana	[illegible]	Feb 8, 2018

Scanned by CamScanner
